# VIO: ontology classification and study of vaccine responses given various experimental and analytical conditions

**DOI:** 10.1186/s12859-019-3194-6

**Published:** 2019-12-23

**Authors:** Edison Ong, Peter Sun, Kimberly Berke, Jie Zheng, Guanming Wu, Yongqun He

**Affiliations:** 1 Department of Computational Medicine and Bioinformatics, University of Michigan, Ann Arbor, MI USA; 2 College of Literature, Science, and the Arts, University of Michigan, Ann Arbor, MI USA; 3 Central Michigan University College of Medicine, Mount Pleasant, MI USA; 4 University of Pennsylvania Perelman School of Medicine, Philadelphia, PA USA; 5 Oregon Health & Science University, Portland, OR USA; 6 Unit of Laboratory Animal Medicine, University of Michigan, Ann Arbor, MI USA; 7 Department of Microbiology and Immunology, University of Michigan, Ann Arbor, MI USA; 8 Center for Computational Medicine and Bioinformatics, University of Michigan, Ann Arbor, MI USA

**Keywords:** Vaccine ontology, Vaccine investigation ontology, Yellow fever vaccine, YF-17D, Vaccine response, LIMMA

## Abstract

**Background:**

Different human responses to the same vaccine were frequently observed. For example, independent studies identified overlapping but different transcriptomic gene expression profiles in Yellow Fever vaccine 17D (YF-17D) immunized human subjects. Different experimental and analysis conditions were likely contributed to the observed differences. To investigate this issue, we developed a Vaccine Investigation Ontology (VIO), and applied VIO to classify the different variables and relations among these variables systematically. We then evaluated whether the ontological VIO modeling and VIO-based statistical analysis would contribute to the enhanced vaccine investigation studies and a better understanding of vaccine response mechanisms.

**Results:**

Our VIO modeling identified many variables related to data processing and analysis such as normalization method, cut-off criteria, software settings including software version. The datasets from two previous studies on human responses to YF-17D vaccine, reported by Gaucher et al. (2008) and Querec et al. (2009), were re-analyzed. We first applied the same LIMMA statistical method to re-analyze the Gaucher data set and identified a big difference in terms of significantly differentiated gene lists compared to the original study. The different results were likely due to the LIMMA version and software package differences. Our second study re-analyzed both Gaucher and Querec data sets but with the same data processing and analysis pipeline. Significant differences in differential gene lists were also identified. In both studies, we found that Gene Ontology (GO) enrichment results had more overlapping than the gene lists and enriched pathway lists. The visualization of the identified GO hierarchical structures among the enriched GO terms and their associated ancestor terms using GOfox allowed us to find more associations among enriched but often different GO terms, demonstrating the usage of GO hierarchical relations enhance data analysis.

**Conclusions:**

The ontology-based analysis framework supports standardized representation, integration, and analysis of heterogeneous data of host responses to vaccines. Our study also showed that differences in specific variables might explain different results drawn from similar studies.

## Background

As one of the most significant inventions in modern medicine, vaccination has been used to efficiently protect humans against many infectious diseases and improve human health. Vaccines are also being developed against cancer [1], allergy [2], and many other non-infectious diseases [3, 4]. However, our efforts to develop vaccines to protect against diseases have not always been successful. The future success of effective vaccine development relies on a deep understanding of protective vaccine-induced immune mechanisms against different diseases. The protective mechanism can be better understood with a systematic analysis of high throughput data being generated in the vaccine domain.

One bottleneck in high throughput vaccine-host interaction studies is that inconsistent experimental results were frequently generated even with similar experimental designs. A typical example is the gene-level host immune responses induced by the live attenuated Yellow Fever vaccine 17D (YF-17D) from various gene expression studies. The live attenuated YF-17D [5] and the sub-strains derived from the original 17D strain [6] are widely used for vaccination against Yellow Fever infections. These vaccine strains can induce strong and effective protective immune responses in vaccinated humans [7, 8]. As a result, YF-17D has become an excellent model to study general host responses induced by vaccinations, and many differentially expressed genes have been reported in YF-17D-vaccinated human subjects. However, these studies reported different results even though similar experimental designs were used. For example, three studies, Gaucher et al. [9], Querec et al. [10], and Scherer et al. [11], all used human subjects who were all vaccinated with YF-17D or YF-VAX (made with a specific YF-17D strain). These three studies generated overlapping but quite different gene expression profiles [9–11].

In our previous study, we systematically classified the conditions in these three studies [9–11] and reported our results in a recent publication [12]. Our study identified approximately 20 variables that existed in a typical vaccine-induced host response investigation study. A large portion of these variables was associated with different values among these three YF-17D vaccine studies [12]. Such variability was likely contributed to the different gene expression profiles observed. Another achievement in the previous study was that we mapped these variables using ontology terms from the Ontology of Biological and Clinical Statistics (OBCS) [13] and the Vaccine Ontology [14–16]. Such ontological modeling facilitated the identification of these variables and the relations among these variables.

Ontology offers an ideal platform to properly and robustly solve the critical issue of different but overlapping results from studies on the same scientific question. Basically, ontology standardizes the representation of entities and relations among entities in a specific domain using human- and computer-interpretable format. Such standardization is important since experimental studies are often reported using inconsistent vocabulary and incomplete representation, often resulting in non-reproducible outcomes. The ontology usage can solve the issues in the standardized experimental and data representation from different studies. Given the nature of ontology, such standardization can also be understood by computers and so useful for data sharing. In addition to standardization, ontology also provides a hierarchical structure and logical relations among different entities, supporting advanced reasoning and data analysis.

The current study extends the previous study as introduced above [12]. Specifically, in this study, we hypothesize that an ontology-based strategy can better analyze (i) different experimental and analysis conditions that significantly affect the gene expression outcomes of host responses to vaccines, and (ii) the conditional gene expression profiles. The first point of the hypothesis can be justified by the phenomenon that human subjects vaccinated with the same Yellow Fever vaccine in three independent studies shown different gene expression profiles based on high throughput transcriptomic profiling. A similar phenomenon has also been observed by other high-throughput studies in various biomedical domains [17–19]. However, the second point of this hypothesis proposes a novel ontology-based strategy that has not been carefully investigated in the field. Although it is commonly known that experimental standardization is critical for reproducible studies, how to use ontology for better understanding the mechanism and underlying knowledge from various studies still poses a big challenge. This paper aims to address this challenge by using the ontology-based vaccine response model system to standardize the experimental conditions and systematically analyze the high throughput data vaccine studies.

Several vaccine investigation-related ontologies exist. The Vaccine Ontology (VO) represents vaccine-related entities, such as vaccines, vaccine components, vaccinations, host responses to vaccines, and the relations among these entities [14–16]. The Ontology of Biological and Clinical Statistics (OBCS) is a community-based ontology of statistics in the biological and clinical domains [13]. The community-based Ontology for Biomedical Investigations (OBI) targets to represent various biomedical investigation components shared by different biomedical communities [20]. Although these ontologies can all be related to vaccine investigation study at some levels, these ontologies are not primarily focused on vaccine investigation and may miss important aspects of vaccine investigation. Ideally, an integrative ontology with a focus on vaccine investigation building on the ontologies mentioned above is needed.

In this study, we first developed a Vaccine Investigation Ontology (VIO), and then applied VIO to systematically and logically model the vaccine investigation process. VIO classifies different variables and the relations among these variables in the vaccine investigation studies. To further address the hypothesis of how different experimental and analysis conditions affect the outcomes of host responses to vaccines, we applied VIO to standardize and analyze the host responses induced by the Yellow Fever vaccine YF-17D and its sub-strains in three studies [9–11].

## Methods

### VIO development and usage

As an extension of the Vaccine Ontology (VO) [14–16], the Vaccine Investigation Ontology (VIO) was developed by following the eXtensible Ontology Development (XOD) principles [21]. Specifically, a list of vaccine investigation-related terms available in VO was initially identified. Ontofox [22] was then used to extract this list of terms and other relevant information (including logical axioms and annotations) from VO, and imported into VIO. Additionally, many OBCS and OBI terms related to vaccine investigation were also imported into VIO using Ontofox [22]. Since VO, OBCS, and OBI all follow the Open Biomedical Ontology (OBO) Foundry ontology development principles [23] and use the same upper-level ontology, Basic Formal Ontology (BFO) [24], these terms coming from different ontologies were efficiently and seamlessly aligned to each other in VIO. The resulting VIO was manually edited and checked using the Protégé OWL editor. The home page and the source code of VIO are publicly available from the GitHub website: https://github.com/vaccineontology/VIO. VIO has been deposited to the Ontobee website: http://www.ontobee.org/ontology/VIO, and BioPortal: http://bioportal.bioontology.org/ontologies/VIO.

In this manuscript, VIO modeling means the usage of the VIO ontology to represent the factors involved in the vaccine investigation process logically. The VIO modeling identified differences among multiple vaccine studies in terms of experimental design and data analysis methods, and helped to explain the different vaccine study outcomes. The Yellow Fever vaccine investigation was used as the specific use case to identify additional terms and rationale for further VIO ontology development.

### Extraction of data from open resources

The NCBI Gene Expression Omnibus (GEO; https://www.ncbi.nlm.nih.gov/geo/) is a web-based public repository that supports the storage of various functional genomics data [25]. The microarray data sets reported in the Gaucher et al. 2008 paper [9] and Querec et al. [10] are available through the GEO under series accession numbers GSE13699 and GSE13486, respectively. The data sets were then extracted from the GEO as instructed in the GEO manual (https://www.ncbi.nlm.nih.gov/geo/info/). The raw data sets from Scherer et al. [11] were not available from GEO or the paper supplemental material files. Therefore, Scherer et al. [11] was excluded from this study.

### Microarray data re-analysis using LIMMA

GEO2R [25] was used to analyze the two microarray datasets as reported in the Gaucher et al. [9] and Querec et al. [10]. In brief, GEO2R applies log2 transformation if the expression values of given GEO dataset are not in log space, and then performs differential expression analysis using Linear Models for Microarray Analysis (LIMMA) [26]. The resulting *p*-values are adjusted for multiple comparisons using the false discovery rate (FDR). The GEO2R results for the two microarray datasets were exported and compared for overlapping using a Venn diagram. The same cut-off (adjusted p-value based on FDR < 0.05 and log fold change less than − 1.3 fold or greater than 1.3 fold) for identifying significant results was applied.

### Gene list comparison studies

Venn diagrams were generated to compare gene lists and identify the shared and unique genes. For the gene-level comparison, gene symbols were updated to official gene symbols using the DAVID Gene ID Conversion Tool (https://david.ncifcrf.gov/conversion.jsp) [27]. All genes analyzed in this study were mapped to their corresponding Entrez Gene IDs using the DAVID Gene ID Conversion. The Gene Ontology (GO) and pathway enrichment analyses of the original study were performed based on the original list of differentially expressed genes. The DAVID bioinformatics resources [27] was used to analyze the similarities and differences of different GO terms enriched in the original analysis or the standardized re-analysis of the two microarray datasets.

The performance of the standardized re-analysis was estimated by the identification of shared significant GO biological processes between the two microarray datasets. The hierarchical structure of significantly enriched GO terms and their related ancestor terms were also visualized and analyzed using GOfox (http://gofox.hegroup.org) [28, 29]. By integrating and extending features from two popular ontology programs Ontofox [22] and Ontobee [30], the GOfox web program is able to generate full or simplified hierarchical GO subsets to classify and display enriched GO terms and their ancestor terms. By considering the multiple inheritances of GO, the GOfox includes a simplified hierarchical classification method that outputs a GO hierarchical structure among enriched GO terms and their minimal upper-level ancestor terms in a user-friendly interactive visualization scheme.

In addition, we also used the Reactome pathway analysis tool [31] to analyze enriched pathways in the Reactome pathway knowledgebase. Both GO biological processes and Reactome pathway enrichment analysis applied adjusted *p*-value based on FDR < 0.05 as the significance cut-offs.

## Results

### VIO ontology development

**Fig. 1:**
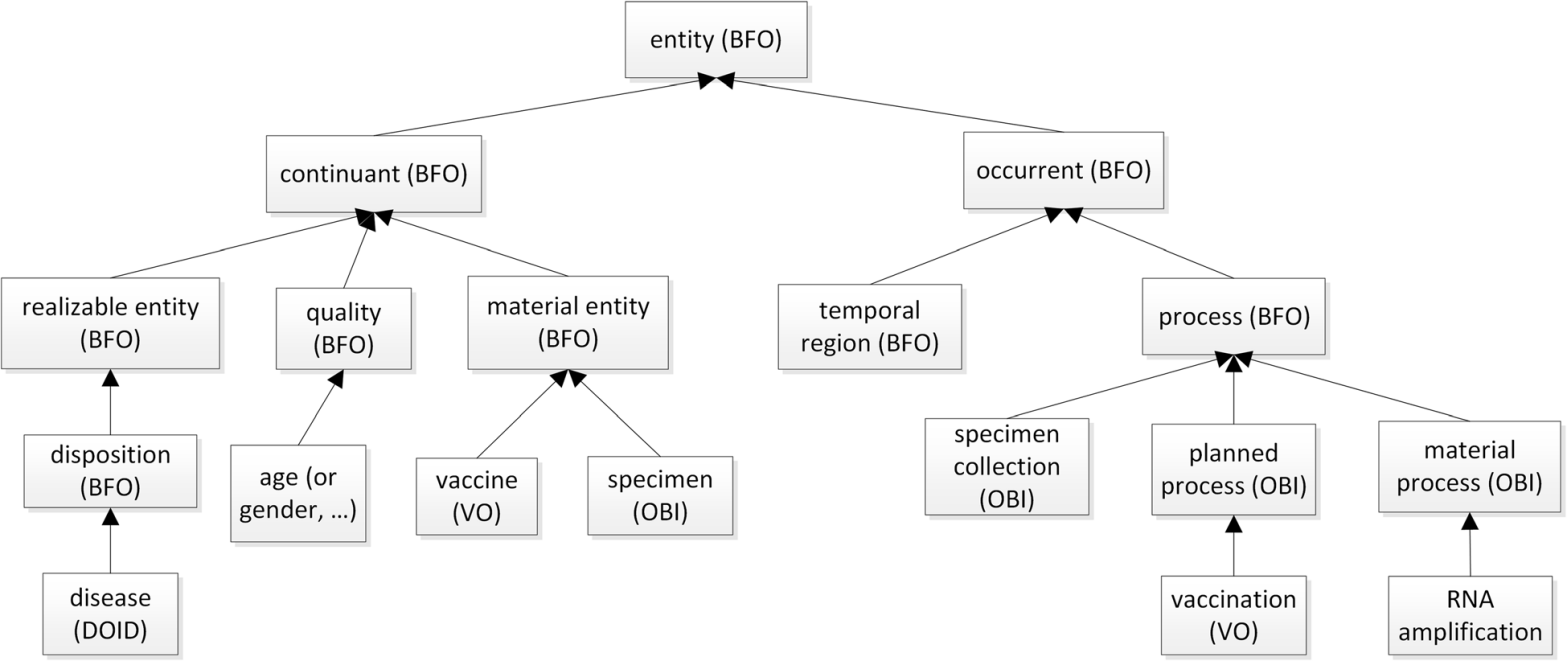
Selected top-level terms and hierarchy of VIO. VIO top-level hierarchy is aligned to the BFO to facilitate data integration

**Fig. 2:**
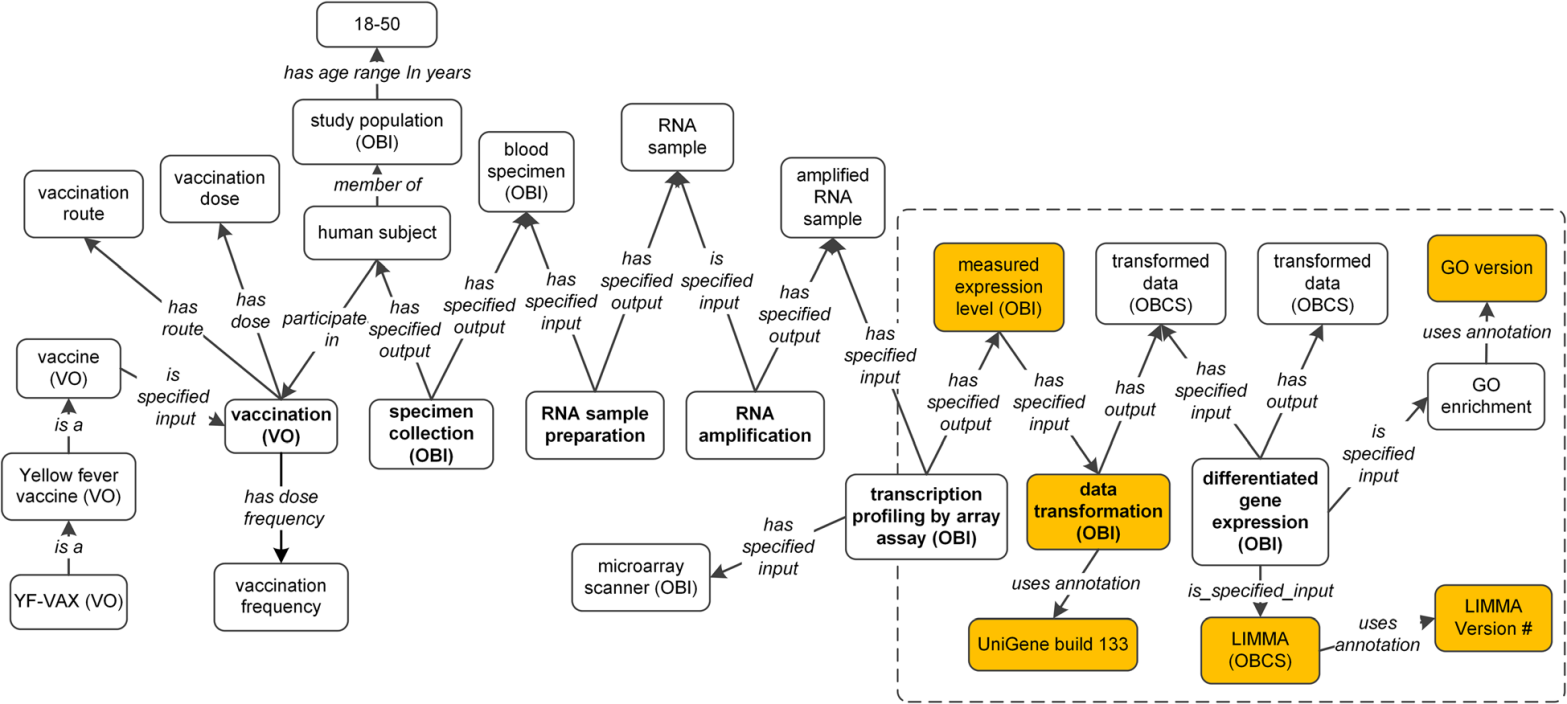
VIO design pattern suitable for representing the YF-VAX vaccination use case. The boxed section includes different components that are all related to data processing and analyses. The brown-colored boxes are examples of variables changeable in our data re-analysis

The top-level hierarchical design of the VIO ontology is shown in Fig. 1. Compared to the VO, VIO focuses on the vaccine investigation, especially on defining and standardizing metadata types in various vaccine investigation studies. Figure 2 was generated to show a representative ontology design pattern, which includes most variables in the three Yellow Fever studies [9–11]. Our modeling of the vaccine experimental investigation identified many variables (e.g., data transformation method, human genome annotation version, significant gene identification method such as LIMMA, LIMMA version, and GO version used for GO enrichment analysis) that are related to data analysis. These variables can be standardized in our data re-analysis process pipeline. On the other hand, the variables for wet-lab experiments were examined because this study focused on the data processing and analysis using ontology-based strategy rather than repeating the experiments with standardized experimental conditions. To a certain extent, studies with different experimental settings can be considered as permutations to the host immune system and can be used to better understand the immune response mechanisms induced by the vaccine immunization. Therefore, controlling these experimental conditions is not necessary to understand the contributions of different variables to the final observed immune response outcomes. Instead, we can carefully dissect and identify the similarities and dissimilarity among these variables from different experimental studies.

Overall, the current version of VIO has 79 classes, 46 object properties, and 44 annotation properties. These VIO terms are obtained from reusing terms from 12 existing ontologies such as VO, OBI, OBCS, and IAO. The detailed VIO ontology statistics can be found at the VIO Ontobee website: http://www.ontobee.org/ontostat/VIO.

### Standardize and re-analyze yellow fever vaccine studies using VIO

The overall hypothesis in our study is that different experimental and analysis conditions significantly affect the analysis result of host responses to vaccines and such conditional gene expression profiles can be analyzed using an ontology-based strategy. A feasible way to test our hypothesis is to re-analyze data sets from different studies. Since data analysis may involve many variables, it needs to standardize these variables using the developed VIO and thus may change the results.

In this work, we tested this hypothesis using the data obtained from previous two Yellow Fever vaccine studies: Gaucher et al. [9] and Querec et al. [10] (Table 1). Our analysis included two scenarios:

(i) *Comparison of statistical analyses of the same data set from a study*. Here we re-analyzed the data set from Gaucher et al. [9], and compared newly analyzed results with the results reported in the published paper [9]. The full gene list from Querec et al. [10] was not available from the publication and thus cannot be studied in this work.
(ii) *Comparison of different data sets from two studies but analyzed with the same statistical design*. We re-analyzed the two datasets available in Gaucher et al. [9] and Querec et al. [10]. Both datasets were extracted from GEO. We used the same statistical method with standardized settings to analyze these two datasets and compare the results.

**Table 1:** Comparison of factors used for the LIMMA analyses of the same data set published in the Gaucher et al. [9]

Factor	Original analysis	Re-analysis
Filtering	Filtered probes with intensity below background	
Normalization	Quantile normalization	
Transformation	Log2 transformation	
Fold change cut-off	< −1.3 or > 1.3	
LIMMA version	Unspecified (before 2008)	LIMMA 3.26.8
LIMMA Software	LIMMA package in Bioconductor	GEO2R
Multiple test correction	False discovery rate (FDR)	
Adjusted *p*-value cut-off based on FDR	0.05	

### Comparison of statistical analyses using the same data set from a study

The original Gaucher et al. paper reported 559 differentially regulated genes in response to Yellow Fever vaccination. The method used in the original paper was via LIMMA data analysis [32]. Using the dataset from Gaucher et al. [9], we re-analyzed the data also using the same LIMMA data analysis method in the GEO2R platform [25]. The purpose of such analytic design was to repeat the analysis method and compare the results between newly analyzed results and the results published in the original paper. Table 1 shows the details of analysis factors used in this comparison.

Overall, there were 554 significantly expressed genes (criteria: adjusted *p*-value based on FDR < 0.05, and log fold change < − 1.3 or > 1.3) in the re-analysis of the dataset obtained from Gaucher et al. [9].

**Fig. 3:**
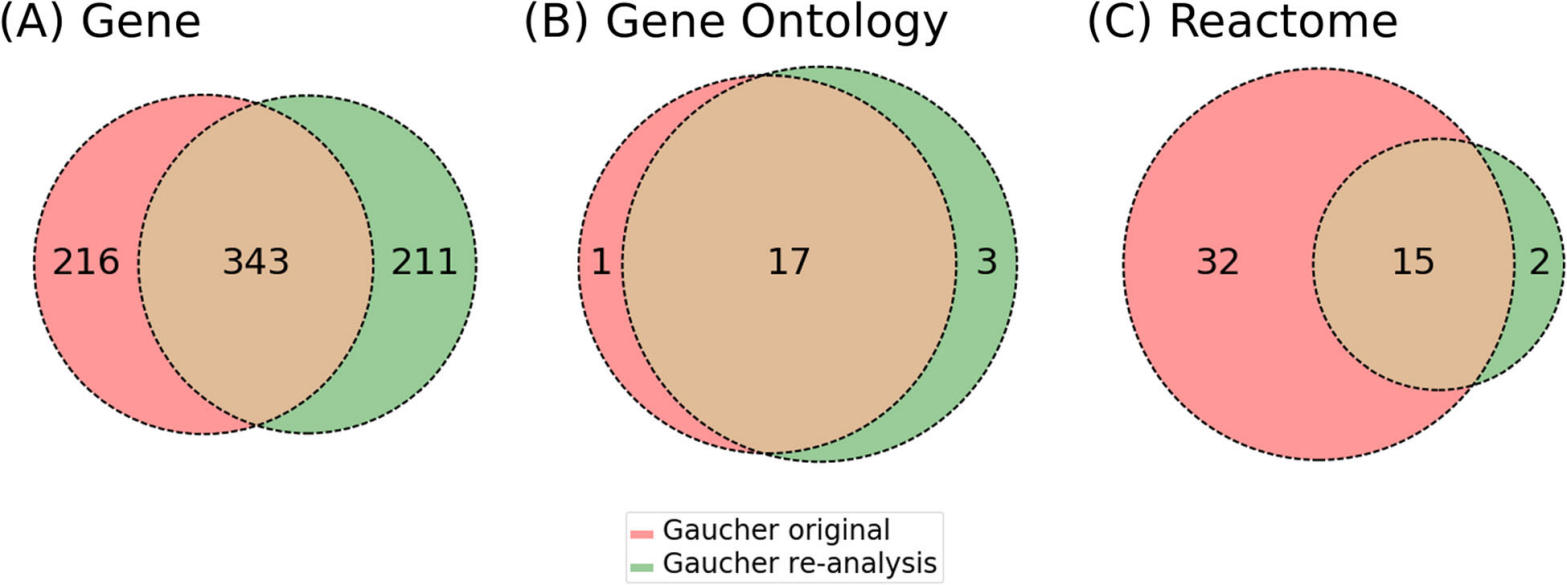
Comparison of the reported result from Gaucher et al. [9] to our re-analysis based on genes, biological process in Gene Ontology and Reactome. Venn diagram illustrating the comparison of significant (adjusted *p*-value based on FDR < 0.05) (**a**) differentially expressed genes, (**b**) Gene Ontology biological process terms, (**c**) Reactome pathways between the original and re-analysis of Gaucher et al. [9]

When we compared Gaucher et al. original results [9] to our re-analysis results, a different set of genes were identified in the re-analysis from those in the original analysis (Fig. 3a). Specifically, comparing the original analysis results in Gaucher et al. paper, our re-analysis found many similarities as well as differences. Although we found 343 shared genes, 211 genes only existed in the re-analysis, and 216 only existed in the original paper [9]. This is likely because the analysis settings can vary, and some of these settings are not clearly specified in the original paper (Fig. 2). More specifically, our VIO-based detailed comparison of analytic conditions (Table 1) showed that only the LIMMA version and the software running the program likely differed. Our re-analysis used the LIMMA version 3.26.8 and was conducted in the GEO2R platform [25]. However, the original LIMMA version was not provided in their publication, and the software appeared to be the LIMMA package in R and Bioconductor [9]. The re-analysis LIMMA version (3.26.8) was released in 2016, which is more recent than the paper publication date (i.e., 2008), suggesting that two different LIMMA versions were applied. Therefore, the VIO modeling of data processing and analysis provides us a way to appropriately explain why the same dataset analyzed with the same LIMMA method resulted in different significant gene lists.

When we compared the enriched GO biological process terms, 17 GO terms were shared between the two analyses, and only 1 GO term from Gaucher original and 3 GO terms from Gaucher re-analysis were unique and not shared (Fig. 3b). Therefore, our study indicated that the GO enrichment study provided us more reproducible results compared to the gene list results.

Furthermore, we performed Reactome-based pathway analyses (Fig. 3c). This study provided another comparison to examine the possible differences at the level of enriched pathways. A total of 15 enriched pathways were shared between two analyses. Only two pathways were enriched in the re-analysis but not in the original analysis.

As shown in this analysis based on the same analytic study and the same dataset, different analysis settings often generated different results. For data re-analysis, the usage of the same data analysis settings is important. In our case, we tried to use exactly the same analysis methods. However, data analysis details are often missing, making it difficult to duplicate the exact same analysis method and setting.

### Comparison of different data sets from two studies but analyzed with the same design

**Fig. 4:**
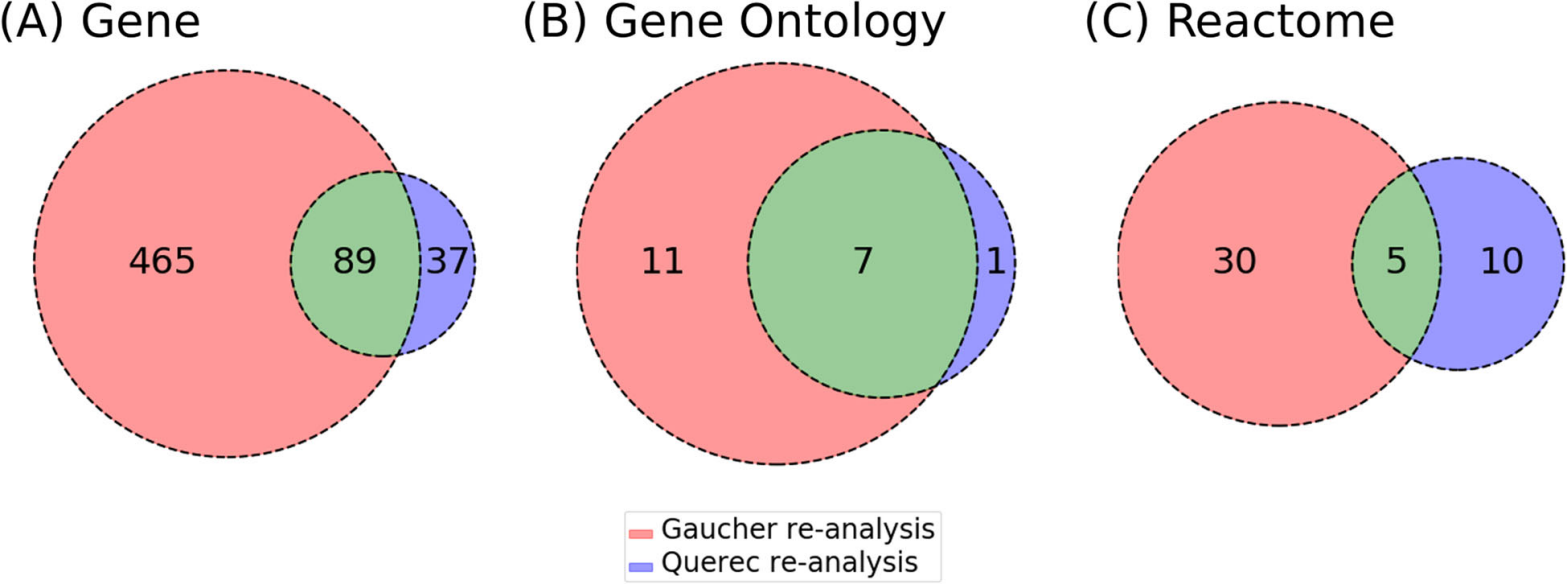
Comparison of the reported result between the re-analysis of Gaucher et al. [9] and Querec et al. [10] based on genes, biological process in Gene Ontology and Reactome. Venn diagram illustrating the comparison of significant (adjusted *p*-value based on FDR < 0.05) (**a**) differentially expressed genes, (**b**) Gene Ontology biological process terms, (**c**) Reactome pathways between the re-analysis of Gaucher et al. and Querec et al.

In this study, we re-analyzed two datasets (Gaucher et al. vs. Querec et al.) using the same data processing setting, the same GEO2R platform, and the same LIMMA data analysis method. Overall, there were 554 and 126 significantly differentiated genes in the re-analysis of Gaucher et al. [9] and Querec et al. [10], respectively (Fig. 4a). In total, 89 genes were shared by these two studies. Meanwhile, 465 significantly differentiated genes were only found in Gaucher re-analysis, and 37 only in Querec re-analysis (Fig. 4a).

Compared to the difference of gene lists between these two studies (Fig. 4a), our re-analysis identified more consistent enriched GO biological processes. As shown in Fig. 4b, after re-analysis, 7 GO terms are shared in both data sets, and only 11 GO terms in Gaucher and 1 in Querec are not shared. This clearly suggested that significantly identified gene lists differ more than the difference in the results of GO enrichment analysis. As seen in the two original studies [9, 10], only four enriched GO biological process terms were shared, and 20 terms were found different. Compared to Fig. 4b, our re-analysis provided more consistent results in terms of the enrichment analysis of GO biological process terms.

**Fig. 5:**
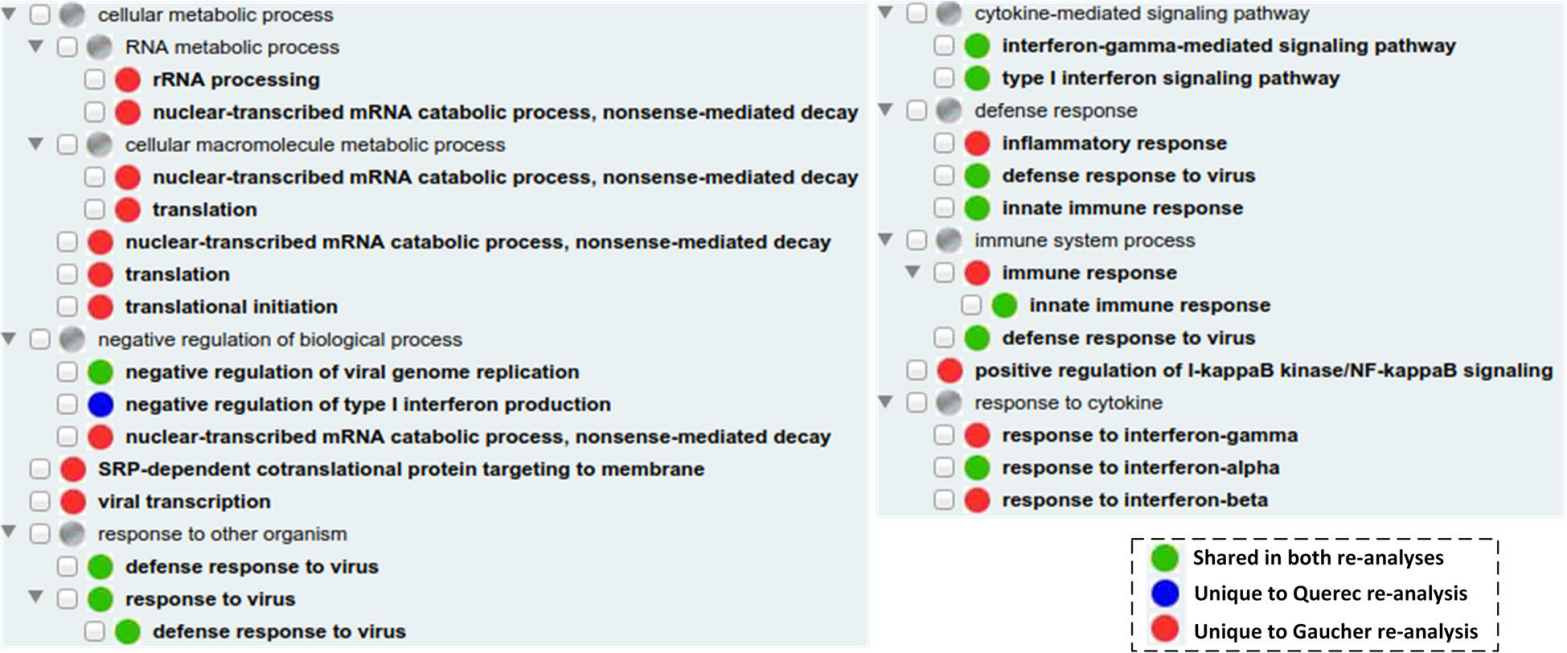
Hierarichal display of significantly enriched GO biological process terms from the re-analysis of Gaucher et al. and Querec et al. using the GOfox tool. Circles colored with green, red, and blue represent GO terms shared in both re-analyses, unique to Gaucher re-analysis, and unique to Querec re-analysis, respectively

It is possible that the non-overlapped GO terms have closer relations in terms of the GO hierarchical structure. For example, these non-overlapped GO terms might share the same parents, siblings, or children terms. To test this hypothesis, we applied the GOfox GO visualization tool [28, 29] to put the enriched GO terms under the context of the GO hierarchical structure (Fig. 5). The shared enriched GO terms (with green color circles) were focused on categories including responses to viruses, cytokine-mediated signaling pathways, and defense response (Fig. 5). Interestingly, responses to three types (alpha, beta, and gamma) of interferon cytokines were identified in the story. The response to interferon-alpha was shared between both re-analyses. However, responses to interferon-beta and interferon-gamma are significantly enriched in only Gaucher re-analysis (with red circles). The only GO term unique to Querec re-analysis was negative regulation of type I interferon production (with blue circle). How different interferon signaling pathways get involved in the protective immunity against Yellow Fever deserves further investigation. Several GO terms under cellular and RNA macromolecule metabolic processes were enriched only in Gaucher re-analysis, suggesting more general metabolic processes were detected in Gaucher re-analysis than Querec re-analysis. This study demonstrated that the hierarchical visualization of the enriched GO terms provided more useful information than plain lists of enriched GO terms.

Our Reactome pathway enrichment analysis found that five enriched pathways were shared in two dataset analyses, and 10 enriched pathways were unique in Querec re-analysis, and 30 unique in Gaucher re-analysis, suggesting more differences (Fig. 4c).

## Discussion

This study focuses on the development of the Vaccine Investigation Ontology (VIO), its application on the identification of different variables in vaccine investigation studies, and the use case demonstration showing the possibility and complexity of using VIO to study different vaccine host response results.

This ontology-based study is novel in that the VIO development and application represent the first ontology effort to standardize and represent vaccine investigation studies from different research studies. Our ontology development followed the state-of-the-art eXtensible Ontology Development (XOD) principles, which support ontology reuse, alignment, design pattern usage, and community extensibility [21]. Our use case study demonstrates how VIO can be used to represent and standardize analytic pipeline of gene expression studies, and such a study can be potentially extended to other biomedical domains.

VIO provides a way to standardize the representation of minimal information standards and metadata representation for vaccine investigations including both experimental and analytic parts. As shown in Fig. 2, VIO can be used to standardize all the variables and metadata types for vaccine investigation studies including the study of host responses to vaccinations. Our VIO modeling identified many variables involved in raw data processing, data transformation, and statistical analyses. We found that although the same LIMMA method was used in the original Gaucher et al. paper [9] and our re-analysis, the results are still quite different (Fig. 3a). By carefully examining all the variables, we found that only specifying the LIMMA method alone is not sufficient to achieve reproducible results. The two LIMMA analyses (original Gaucher et al. [9] and our re-analysis) using the same dataset generated inconsistent results due to two different software versions. This emphasized the use of VIO to model and represents various metadata types to ensure robust and reproducible studies. The VIO modeling can also recommend what metadata types should be provided by the authors for making analysis reproducible.

It is interesting to observe that although the gene lists from our two standardized scenarios using the same dataset differed a lot, the GO enrichment results were more consistent between groups of studies (Fig. 3b and Fig. 4b). This suggested that although the specific significantly differentiated genes might differ given different conditions, they participate in similar or related biological processes. Furthermore, our GOfox analyses showed that even the GO terms might show differences, the hierarchical structure comparison between the two sets of results showed that the different GO terms could often be aligned under the same ancestor GO terms. The identification of these hierarchical structures makes it better to understand the underlying molecular mechanisms.

When the enrichment results of GO biological processes and Reactome pathways are compared, the GO enrichment results show more overlapping than the Reactome pathway enrichment results. This suggests that GO biological processes are broader and more inclusive than the Reactome pathways in this scenario. It is possibly due to the fact that Reactome pathway annotations have lower coverage of human proteins than GO annotations: close to 11,000 proteins in Reactome (https://www.reactome.org/about/statistics) vs. over 17,000 proteins in GO (http://current.geneontology.org/annotations/) based on releases in 2018, resulting from different annotation criteria. More future analyses are deserved and required to study the detailed gene products, GO terms, and pathways to identify the underlying mechanisms and uncover new scientific insights.

Different from this study where many data processing and analysis-related variables exist, a previous meta-analysis of *Brucella* vaccine protection study shows only one data-related variable (i.e., protection or not) [33]. The *Brucella* meta-analysis study focuses on the effects of different experimental conditions toward the same vaccine protection efficiency. In that case, the data analysis is simple, but the roles of different experimental conditions can be determined. In total, the *Brucella* vaccine protection study identified approximately 20 experimental variables whose variations may change the protection outcomes. One major difference between these two types of vaccine investigations is that *Brucella* vaccine protection study includes a step of virulent pathogen challenge, while the Yellow Fever vaccine study does not have the challenge step. We plan also to use VIO to model the vaccine protection studies and make VIO useful in standardized vaccine protection studies.

The VIO ontology development is an ongoing project. We will continue to develop VIO to cover more experimental conditions such as genetic variations and apply VIO to test different study scenarios using the ontology-based strategy described in this paper. The genetic variations among experimental subjects may also affect the outcomes of vaccine investigation studies. Although our cases have so far not investigated the effect of host genetic variations, we plan to include various types of genetic variations in our future VIO development to address the importance of genetic variations in vaccine outcome studies. As a result, we will extend the VIO ontology-based methods to study more datasets and address specific scientific questions. For example, ImmPort (http://www.immport.org/) is the NIH-funded bioinformatics repository for the field of immunology [34]. ImmPort stores a large volume of vaccine-induced host immune response data. It is difficult to systematically process and analyze the heterogeneous data types obtained from different experimental studies and groups. However, the VIO ontology framework generated in this article provides a good strategy to tackle this problem.

Not only in the vaccine domain, the challenge of standardizing and integrating homogenous data also exists in other biomedical domains, and can be caused by experimental or analytical factors in the metadata. For example, the fields of cancer prognosis and prediction [35], stem cell differentiation and aging [36], lung disease [37] all face the challenge. There are various sources of errors and inconsistencies associated with different high throughput technologies such as the microarray technology [38], flow cytometry [39], and RNA-seq [40]. This study represents an effective ontology-based effort to solve the critical issue of different but overlapping results from studies on the same scientific question. In addition, we have developed VIO by following the state-of-the-art strategy and ensure that the ontology is open and logically well-formed to enable interoperability to ontologies in other biomedical domains. The interoperability can further solve the critical issue of data heterogeneity and inconsistency in interdisciplinary studies. The strategy demonstrated in our VIO work can be further extended to solve the similar problems in other research domains.

## Conclusions

In summary, the development of VIO and its application on standardizing and analyzing vaccine investigation study helps to better integrate and understand the underlying immune mechanism induced by vaccination. The experimental investigations following the VIO modeling can also improve or ensure the reproducibility of experimental and data analysis. This study provides a demonstration of the ontology-based strategy is feasible to be applied to other biomedical domains.

## Data Availability

The source code of VIO is freely available at the VIO GitHub website: https://github.com/vaccineontology/VIO. VIO is also deposited in the Ontobee [30] ontology repository: http://www.ontobee.org/ontology/VIO.

## Abbreviations

BFO: Basic Formal
Ontology GEO: NCBI Gene Expression Omnibus
GO: Gene Ontology
LIMMA: Linear Models for Microarray Analysis
OBCS: Ontology of Biological and Clinical Statistics
OBI: Ontology of Biomedical Investigations
VIO: Vaccine Investigation Ontology
VO: Vaccine Ontology
YF-17D: Yellow Fever vaccine 17D
